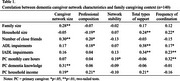# Examining Variations in Dementia Caregiver Network Structure and Professional Composition

**DOI:** 10.1002/alz70858_097631

**Published:** 2025-12-24

**Authors:** William McConnell, Katherine Haggar

**Affiliations:** ^1^ Florida Atlantic University, Boca Raton, FL, USA

## Abstract

**Background:**

Recent research suggests that persons living with dementia (PLWD) rely on complex networks of interconnected informal and professional caregivers. However, little is known about the structure and composition of caregiver networks and few studies address both informal and professional caregivers simultaneously. This study presents a detailed social network analysis of mixed informal and professional caregiver networks supporting PLWD.

**Method:**

A survey‐based interview protocol was designed to measure caregiver networks using 10 name generators that identify all participants in caregiving tasks. We obtained information on caregiver network characteristics (size, composition, stability, supportiveness, care coordination) and family caregiving context (family size, household size, social support, disease severity, primary caregiver background). We administered the protocol to primary caregivers for PLWD utilizing outpatient services at a memory disorder clinic. Bivariate statistics were used to identify significant associations between caregiver network characteristics and family contextual factors.

**Result:**

176 primary caregivers (age 28‐91) for PLWD provided information on 1,566 caregiver network members; thus, PLWD were supported by large networks of 8.9 caregivers, on average. Caregiver networks were composed of 46% informal caregivers and 54% professionals. Caregiver network characteristics varied based on family caregiving context (Table 1). PLWD with larger families had larger caregiver networks (*r* = .28; *p* < .01) but not necessarily more stability, support, or care coordination. Household size, in contrast, was associated with relatively fewer professional caregivers (*r* = ‐.19; *p* < .05) but more types of support (*r* = .24; *p* < .01) and more frequent coordination (*r* = .22; *p* < .05). Only 45% of PLWD's family members participated in caregiving tasks compared to 72% of household members. Disease severity (ADLs) was associated with more professional caregivers (*r* = .18; *p* < .05), more support (*r* = .38; *p* < .01) and more frequent coordination (*r* = .17; *p* < .05). Primary caregiver resources mattered: household income was associated with larger caregiver networks (*r* = .19; *p* < .05), more professional caregivers (*r* = .21; *p* < .05), and more support (*r* = .21; *p* < .05), but not necessarily stability or coordination.

**Conclusion:**

We found that PLWD are supported by large networks of informal and professional caregivers. Moreover, caregiver networks adapt to family caregiving context. Future studies should investigate consequences of caregiver network variations for caregiving‐related outcomes like quality and costs of care, formal services use, and caregiver burden.